# Interpreting the Results of Trials of BCG Vaccination for Protection Against COVID-19

**DOI:** 10.1093/infdis/jiad316

**Published:** 2023-08-10

**Authors:** Christie C A Noble, Nicole L Messina, Laure F Pittet, Nigel Curtis

**Affiliations:** Department of Paediatrics, The University of Melbourne, Parkville, Victoria, Australia; Infectious Diseases Research Group, Murdoch Children's Research Institute, Parkville, Victoria, Australia; Department of Paediatrics, The University of Melbourne, Parkville, Victoria, Australia; Infectious Diseases Research Group, Murdoch Children's Research Institute, Parkville, Victoria, Australia; Department of Paediatrics, The University of Melbourne, Parkville, Victoria, Australia; Infectious Diseases Research Group, Murdoch Children's Research Institute, Parkville, Victoria, Australia; Paediatric Infectious Diseases Unit, Faculty of Medicine, Geneva University Hospitals, Geneva, Switzerland; Department of Paediatrics, The University of Melbourne, Parkville, Victoria, Australia; Infectious Diseases Research Group, Murdoch Children's Research Institute, Parkville, Victoria, Australia; Infectious Diseases, The Royal Children's Hospital Melbourne, Parkville, Victoria, Australia

**Keywords:** BCG vaccination, COVID-19, SARS-CoV-2, off-target effects

## Abstract

BCG vaccination has beneficial off-target (“nonspecific”) effects on nonmycobacterial infections. On this premise, trials set out to investigate whether BCG provides off-target protection against coronavirus disease 2019 (COVID-19). A literature search identified 11 randomized “BCG COVID-19” trials, with conflicting results. These trials and the differences in their study design are discussed using the PICOT (participants, intervention, control, outcome, and timing) framework to highlight the factors that likely explain their inconsistent findings. These include participant age, sex and comorbid conditions, BCG vaccination strain and dose, outcome measure and duration of follow-up. Understanding how to control these factors to best exploit BCG's off-target effects will be important in designing future trials and intervention strategies.

## Teaser

Eleven randomized trials have reported the effect of BCG vaccination on coronavirus disease 2019, with conflicting results. Variation in study design likely explains the inconsistent findings, including differences in participant demographics, BCG vaccination strategy, outcome measure and follow-up duration.

The recent global pandemic focused attention on the potential for BCG vaccination to provide protection against coronavirus disease 2019 (COVID-19) [1, 2]. The World Health Organization recommends universal BCG vaccination after birth in regions with a high incidence of tuberculosis, primarily to provide infants with specific protection against disseminated tuberculosis [3]. However, BCG vaccination also has beneficial off-target (“nonspecific”) effects on other infections [4, 5]. These effects are proposed to be mediated by BCG-induced immunomodulation, including functional reprogramming of the innate immune system, termed “trained immunity” [6, 7].

BCG's off-target effects may be evident only in certain circumstances. Some studies have shown benefits of BCG vaccination in reducing all-cause mortality and infection in infants and children [8–10], while others have shown limited protection or no effect [11–13].

**Figure 1.**
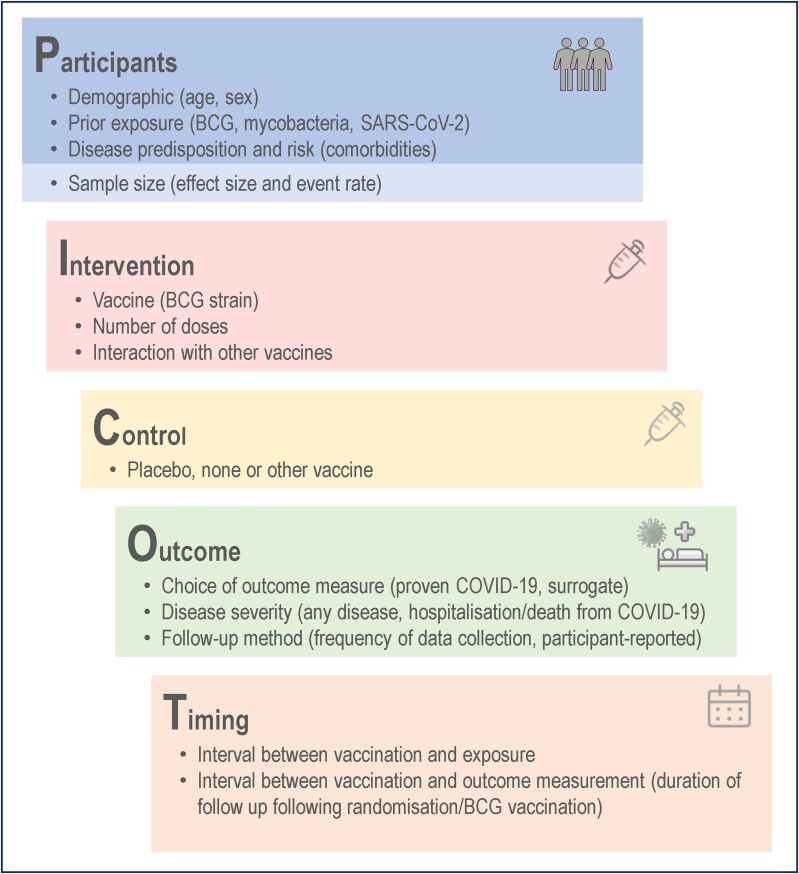
Features of study design to be considered using the PICOT (participants, intervention, control, outcome, and timing) framework, as applied to the trials of BCG vaccination for protection against coronavirus disease 2019 (COVID-19). Abbreviation: SARS-CoV-2, severe acute respiratory syndrome coronavirus 2.

This review summarizes the results of randomized trials of BCG vaccination for protection against COVID-19 or its impact (“BCG COVID-19 trials”). It discusses potential explanations for the contradictory findings in these and other trials of BCG's off-target effects. These are addressed in the context of study design, exemplified by the PICOT framework [14, 15], namely, those relating to participants, intervention, controls, outcome measures, and time of follow-up (Figure 1).

## METHODS

**Figure 2.**
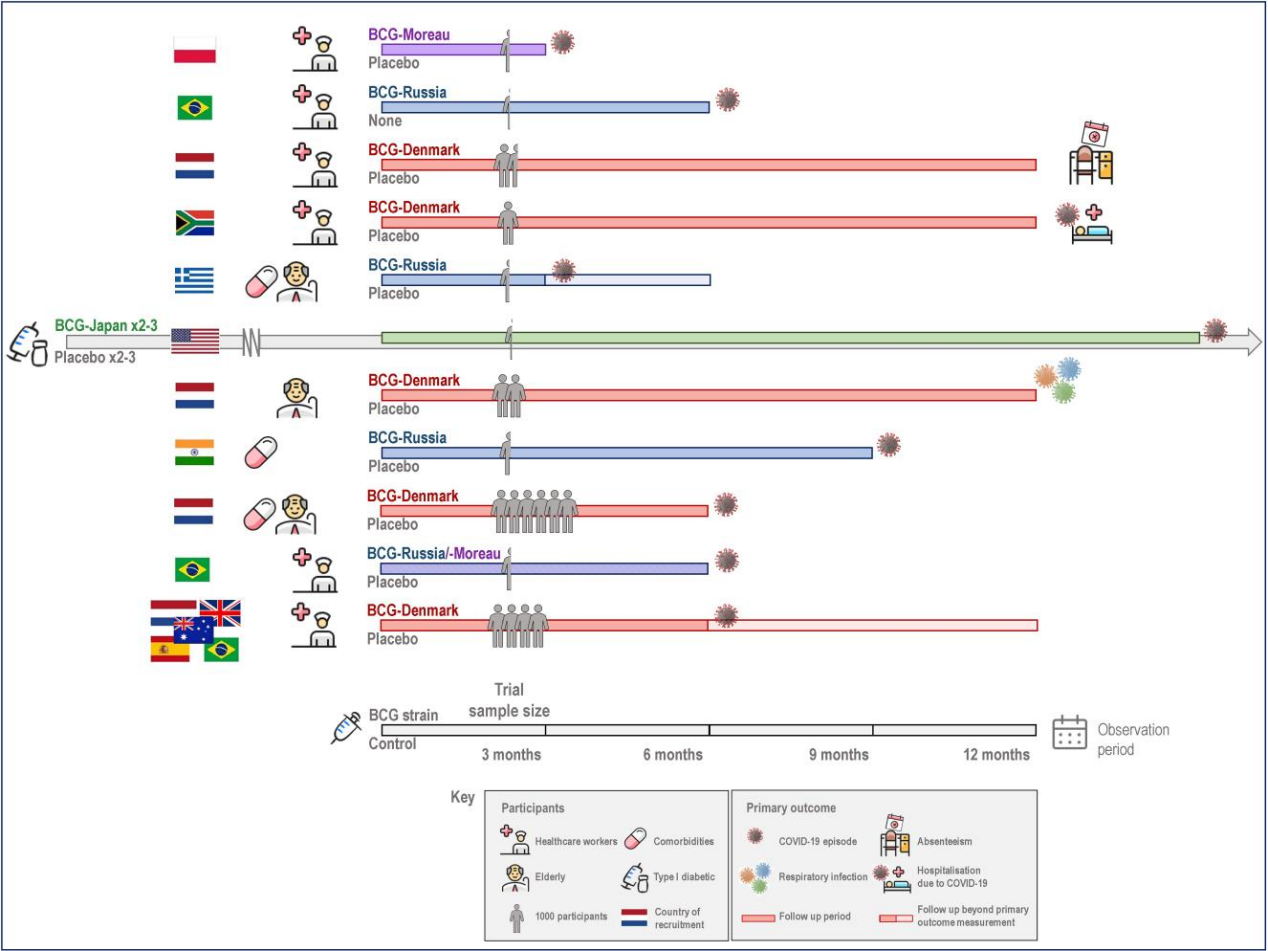
Study design of trials of BCG vaccination for protection against coronavirus disease 2019 (COVID-19). A summary of the 11 trials, highlighting the variation in their study design. Ten trials were designed specifically to investigate the effect of BCG on COVID-19. The US trial was a parallel study, using participants in an existing trial investigating the effect of multiple BCG vaccination doses on glycemic control in type 1 diabetes mellitus (depicted by the longer arrowed bar).

A literature search revealed 11 published trials addressing the effect of BCG on COVID-19 [16–26], with a number of trials remaining unpublished (Table 1, Figure 2, Supplementary Tables 1*A,* 1*B,* 2, and 4, and Supplementary Figure 1). Further details are provided in the Supplementary Methods.

**Table 1.** Details of 11 Published Trials of BCG Vaccination for Protection Against Coronavirus Disease 2019

Location; Trial Name; Authors	Participant Details (Previous BCG Vaccine Rate)	Total No. Enrolled (Randomization Ratio)	Intervention: BCG Strain	Control Intervention	Follow- up Duration	Primary Outcome^a^	Cases or Days, No. (%)		Reported Summary Measure
							BCG Group	Control Group	
Poland; “Polish trial”; Czajka et al [16]	HCWs aged >25 y (100% presumed)	354 (1:1)	Poland BCG-10	Saline	3 mo	COVID-19 cases (PCR confirmed)	**38** **(22.6)**	44 (25.3)	“No significant correlation”
Brazil; “Brazilian revaccination trial”; Dos Anjos et al [17]	HCWs with previous BCG (100% confirmed)	138 (1:1)	Russia 361-I, SII	None	180 d	COVID-19 cases (PCR/serology confirmed)	**8** **(12.5)**	11 (16.4)	“Did not reach statistical significance”
Netherlands; BCG-CORONA; Ten Doesschate et al [20]	HCWs (17%)	1511 (1:1)	Denmark 1331, SSI	Saline	Median, 358 d	Duration of unplanned absenteeism for any illness, including quarantine (% of planned workdays)	**3443 d** **(2.8)**	3298 d (2.7)	Adjusted risk ratio, 0.94 (95% CrI, .78–1.15)
						Secondary outcome: COVID-19 cases (symptomatic and PCR/RAT confirmed)	**102** **(14.2)**	108 (15.2)	Adjusted HR 0.94 (95% CI .72–1.13)
South Africa; “South African trial”; Upton et al [22]	HCWs (100% presumed)	1000 (1:1)	Denmark 1331, SSI	Saline	52 wk	Hospitalization due to COVID-19	10 (2)	**5** **(1)**	HR 2.0 (95% CI, .69–5.9)
						Secondary outcome: COVID-19 cases (PCR/antigen confirmed)	98 (19.6)	**92** **(18.4)**	HR, 1.08 (95% CI, .82–1.42)
Greece; ACTIVATE-2; Tsilika et al [21]	Adults aged ≥50 y with comorbid conditions (unknown)	301 (1:1)	Russia 361-I, SII	Saline	3 mo/6 mo	COVID-19 cases (combination of “possible,” “probable,” and “definitive”symptoms and/or molecularly confirmed) in first 3 mo	**2** **(1.4)**	10 (6.5)	*P* = .09
USA (existing trial); “US trial”; Faustman et al [26]	Type 1 diabetes mellitus (0%)	144 (2 BCG: 1 placebo)	Japan Tokyo-172 (≥3 doses)	Saline	15 mo	COVID-19 cases (symptomatic and serology ± PCR confirmed)	**1** **(1.0)**	6 (12.5)	Vaccine efficacy, 92% (*P* = .006)
Netherlands; BCG-CORONA-ELDERLY; Moorlag et al [18]	Adults aged ≥60 y (27%)	2014 (1:1)	Denmark 1331, SSI	Saline	12 mo	Clinically relevant respiratory tract infections requiring medical intervention	29 (2.9)	**24** **(2.4)**	Subdistribution HR, 1.26 (98.2% CI, .65–2.44)
						Secondary outcome: COVID-19 cases (PCR confirmed)	51 (5.1)	**48** **(4.8)**	Subdistribution HR, 1.05 (95% CI, .71–1.56)
India; BRIC; Sinha et al [19]	Adults aged 18–60 y with comorbid conditions (unknown)	495 (1:1)	Russia SII	Saline	9 mo	COVID-19 cases (CB-NAAT-confirmed)	18 (7.3)	**17** **(6.8)**	OR, 1.08 (95% CI, .54–2.14)
						COVID-19 cases (“probable”; symptomatic only)	**15** **(6.1)**	36 (14.5)	OR, 0.38 (95% CI, .20–.72)
Netherlands; BCG-PRIME; Koekenbier et al [24]	Adults aged ≥60 y with comorbid conditions (15%)	6112 (1:1)	Denmark	Saline	6 mo	COVID-19 cases (symptomatic and PCR/antigen/imaging confirmed)	129 (4.2)	**115** **(3.8)**	Subdistribution HR, 1.12 (95% CI, .87–1.44)
Brazil; ProBCG; Santos et al [25]	HCWs (93%)	278 (1:1)	Moreau/Russia SII	Saline	6 mo	COVID-19 cases (PCR/serology confirmed)	**12** **(0.52)** ^b^	17 (0.75)^b^	HR, 0.65 (95% CI, .31–1.39)
Australia, UK, Brazil, Netherlands, Spain; BRACE; Pittet et al [23]	HCWs (77%)	3988 (1:1)	Denmark 1331, AJV	Saline	6 mo/12 mo	COVID-19 cases (symptomatic and PCR/RAT/serology confirmed) in first 6 mo	132 (14.7)^c^	**106** **(12.3)^c^**	Adjusted probability difference, +2.4% (95% CI −.7%–5.5%)
						Severe COVID-19 cases (hospitalized/nonhospitalized) in first 6 mo	75 (7.6)^c^	**61** **(6.5)^c^**	Adjusted probability difference, +1.1% (95% CI, −1.2% to 3.5%)

Bold text has been used to indicate the group with the more favourable outcome.

Abbreviations: AJV, AJ Vaccines; CB-NAAT, cartridge-based nucleic acid amplification test; CI, confidence interval; COVID-19, coronavirus disease 2019; CrI, credible interval; HCWs, healthcare workers; HR, hazard ratio; OR, odds ratio; PCR, polymerase chain reaction; RAT, rapid antigen test; SII, Serum Institute of India; SSI, Statens Serum Institute.

^a^Secondary outcomes are noted for trials in which COVID-19 cases were not reported as the primary outcome.

^b^Incidence as reported by the trial, based on person-days of observation.

^c^Adjusted estimated percentage in 6 months (adjusted for stratification factors used at randomization: age, geographic location, and presence of comorbid conditions).

## RESULTS

### Trial Design Overview

Ten trials recruited participants in a single country, and 1 trial recruited in 5 countries [23]. The trials followed between 138 and 6112 participants, who were healthcare workers [16, 17, 20, 22, 23, 25], elderly [18, 21, 24] or high risk (with type 1 diabetes mellitus [26] or a significant comorbid condition [19]), for periods of 3–15 months between January 2020 and May 2022. Ten used a single dose of BCG (strains included Denmark [18, 20, 22–24], Moreau [16, 25], and Russia [17, 19, 21, 25]), while the 11th administered at least 3 doses of BCG-Japan over a 2-year period before the parallel COVID-19 study [26]. Rates of previous BCG vaccination varied between 0% and 100%. Ten trials were placebo controlled and double-blinded; 1 was single-blinded with no intervention in the control group [17].

Eight trials reported the incidence of COVID-19 as a primary outcome, with varying definitions. The other 3 reported hospitalization due to COVID-19 [22], absenteeism due to any illness [20] or any respiratory tract infections requiring medical attention [18] as primary outcomes, with COVID-19 incidence as a secondary outcome.

### Results of Primary Outcomes

Six trials reported a beneficial effect of BCG vaccination on their primary outcome (Table 1 and Supplementary Table 1*B*). The largest beneficial effects were reported by the Greek ACTIVATE-2 trial (fewer cases of combined possible/probable/definitive COVID-19 at 3 months) [21] and the US study in type 1 diabetics (reduced symptomatic severe acute respiratory syndrome coronavirus 2 [SARS-CoV-2]–confirmed episodes) [26]. The Polish trial and 2 Brazilian trials (Brazilian revaccination trial and ProBCG) reported a lower proportion of COVID-19 cases in the BCG group, and the Dutch BCG-CORONA trial reported less unplanned absenteeism in the BCG group, all with weak evidence [16, 17, 20, 25].

In contrast, 4 trials reported a negative effect of BCG on their primary outcome, all with weak evidence. The South African trial reported that 10 of 15 hospitalizations due to COVID-19 occurred in the BCG group [22]. The Dutch BCG-CORONA-ELDERLY and BCG-PRIME trials reported more respiratory tract infections requiring medical intervention and COVID-19 cases, respectively, in the BCG group [18, 24]. In the international BRACE trial, there were more episodes of both symptomatic and (combined hospitalized and nonhospitalized) severe COVID-19 in the BCG group [23]. Finally, the Indian BRIC trial reported mixed results for their split primary outcome, with no clear difference in molecularly confirmed COVID-19 cases but fewer probable COVID-19 cases, based on symptom screening, in the BCG group [19].

### Results of Secondary Outcomes Related to COVID-19

#### COVID-19 Incidence

Cases of COVID-19 were similar between groups in all 3 trials reporting COVID-19 incidence as a secondary outcome, with only weak evidence of differences (Table 1) [18, 20, 22].

#### Severe COVID-19 and Mortality

There were generally few severe COVID-19 episodes (Supplementary Table 2). Three trials reported fewer hospitalizations due to COVID-19 in the BCG than in the placebo group: ACTIVATE-2 (2 vs 6, respectively; *P* = .28) [21], BRIC (0 vs 6; *P* = .03) [19] and BCG-PRIME (18 vs 21; sub-distribution hazard ratio 0.86 [95% confidence interval, .46–1.61]) [24]. Although the BRACE trial reported more severe COVID-19 events in the BCG group, this difference was attributable to nonhospitalized severe disease, with 5 hospitalized cases in both groups [23]. The South African trial reported more hospitalizations due to COVID-19 in the BCG group (and more participants with severe symptoms), although 2 deaths due to COVID-19 occurred in the placebo group [22]. There were a small number of COVID-19–related hospitalizations in 3 further trials, with no clear trend between groups [17, 18, 20].

In addition to the South African trial, 4 other trials reported deaths related to COVID-19: BCG-PRIME reported 5 in the BCG group and 6 in the placebo group [24], the BRIC and BRACE trials reported 1 death in the placebo group [19, 23] and BCG-CORONA-ELDERLY reported 1 death in the BCG group [18]. Three deaths were reported in the ACTIVATE-2 trial (all in the placebo group), but it was not stated whether these were COVID-related [21]. Deaths due to other causes are shown in Supplementary Table 2.

## DISCUSSION

### Participants

#### Age

BCG vaccination is likely to have different effects throughout the life course (in neonates and infants [27–29], in children and adults [30], and in elderly adults [31]). The strongest evidence for BCG's beneficial off-target effects comes from studies in neonates and young infants, whose developing immune systems may be more susceptible to the immunomodulatory effect of BCG vaccination [8]. BCG-induced trained immunity is well described in adults [32–34], but there is less evidence that this translates to significant clinical effects. The exception may be in elderly persons, who are also vulnerable to infection owing to altered innate immunity and immunosenescence [35] and in whom clinical protection against respiratory and other infections after BCG vaccination has been reported [36].

Three of the BCG COVID-19 trials specifically recruited older age groups (>50 years in the ACTIVATE-2 trial and >60 years in the BCG-CORONA-ELDERLY and BCG-PRIME trials) [18, 21, 24] (Supplementary Table 1*A*). Although recruiting participants of similar age, these 3 trials had multiple other differences in study design.

The ACTIVATE-2 trial reported fewer possible/probable/definitive COVID-19 cases in the BCG group at 3 months [21]. This finding was not replicated in the BCG-CORONA-ELDERLY trial (higher incidence of clinically relevant respiratory tract infections and COVID-19 cases in the BCG group) [18] or in BCG-PRIME (higher incidence of COVID-19 cases in the BCG group) [24]. There were slightly more COVID-19­–related hospitalizations in the control group in all 3 trials.

Two further trials, both in healthcare workers, reported subgroup analysis by age category, with neither reporting strong evidence for a difference between age groups in the effect of BCG [20, 23]. In the BRACE trial, the mean adjusted duration of COVID-19 symptoms was lower in the BCG group in all age groups, but the difference was largest in the oldest age group (≥60 years) [23].

#### Sex

Sex is well recognized to influence immune responses [37, 38]. Similarly, trials and observational studies suggest that off-target effects of vaccines, including BCG, are influenced by sex [10, 39–41]. The proportion of female participants in the 11 trials varied between 32% and 81% (Supplementary Table 1*A*). The 6 trials in healthcare workers had a predominance of female participants [16, 17, 20, 22, 23, 25]. There was no obvious trend in the effect of BCG on COVID-19 outcomes in the trials with a female or male predominance. The effect of sex was reported in subgroup analysis by 3 trials, none of which reported strong evidence for a difference between male and female participants in the effect of BCG on their primary outcome [17, 18, 23] (see the Supplementary Discussion).

#### Predisposition and Risk

The beneficial clinical effects of BCG may only be apparent when there is sufficient risk in terms of disease burden or host predisposition. The strongest evidence for BCG's off-target effects on infections and all-cause mortality in young infants has been in high-mortality settings. A randomized trial in Uganda [10] reported reduced infections in the first 6 weeks of life in BCG-vaccinated infants, but similar trials in Denmark [13] and Australia [12] showed minimal impact of BCG on infections. Notably, the effect of neonatal BCG vaccination on atopic eczema was greatest in “high risk” infants in the Danish and Australian trials [42–44].

In the context of COVID-19, this principle might be evident in the protection of participants at increased risk of severe disease, such as those of older age or with significant comorbid conditions. Four of the BCG COVID-19 trials specifically recruited adults with comorbid conditions (Supplementary Table 1*A*), including adults aged <60 [19], >50 [21], or >60 years [24] with a significant comorbid condition or <50 years with type 1 diabetes mellitus [26].

Of these 4 trials, 2 reported a beneficial effect of BCG vaccination on their measure of COVID-19 incidence (symptomatic and confirmed in the US trial [26] and combined possible/probable/definitive in the ACTIVATE-2 trial [21]), while BCG-PRIME reported a higher incidence of symptomatic COVID-19 in the BCG group [24]. The BRIC trial reported that BCG had a considerable beneficial effect on probable COVID-19 (symptoms only) but minimal effect on confirmed COVID-19 cases [19]. The BRIC, ACTIVATE-2, and BCG-PRIME trials reported a lower incidence of COVID-19–related hospitalization in the BCG group, although the absolute differences were small (Supplementary Table 2) [19, 21, 24].

Two further trials reported subgroup analyses by comorbid condition [18, 23]. The BCG-CORONA-ELDERLY trial found no evidence that comorbid conditions modified BCG's effect on respiratory infections or COVID-19 incidence [18]. The BRACE trial reported weak evidence that the negative impact of BCG vaccination on both symptomatic and severe (hospitalized and nonhospitalized) COVID-19 was greater in participants with certain comorbid conditions [23]. In summary, there was not strong evidence for an impact of vulnerability (age and comorbid conditions) on the effect of BCG vaccination on COVID-19.

#### Prior BCG/Mycobacterial Exposure

Previous exposure to mycobacteria or BCG vaccination may alter the off-target effects of BCG [45, 46]. Interferon-γ release assay testing at baseline showed that almost half of participants in South Africa [22], 13% in ProBCG [25], and 12% of Brazilian participants in the BRACE trial [23] had latent tuberculosis infection (Supplementary Table 1*A*). Two other trials did tuberculin skin testing at baseline and excluded participants with a positive result [16, 21]. None of the trials reported subgroup analysis by interferon-γ release assay or tuberculin skin test result.

Prior BCG vaccination rates varied between trials (Supplementary Table 1*A*). Three trials assessed BCG revaccination only, although a variable proportion of participants had an existing BCG scar [16, 17, 22]. In 2 trials, previous BCG vaccination was not confirmed with participants but likely due to historical BCG programs [19, 21]. In 5 further trials, participant report of previous BCG vaccination ranged from 15% to 93% [18, 20, 23–25], while the remaining trial excluded BCG-vaccinated participants [26].

Of the 4 trials reporting subgroup analyses, none found strong evidence of an interaction between previous BCG and the effect of BCG vaccination [18, 20, 23, 24]. Two trials investigated the impact of existing BCG scar(s) on the effect of BCG revaccination and found no evidence of an interaction [16, 17].

#### Prior SARS-CoV-2 Infection

Testing participants for prior SARS-CoV-2 infection at baseline clarified whether trials were testing protection against primary infection, or also the boosting of memory responses to protect against reinfection. Six trials excluded participants with positive baseline serology, with [19, 23, 25] or without [16, 17, 21] additional SARS-CoV-2 antigen testing. A seventh trial included the 15% of participants with positive baseline serology [22]. Three trials did not do baseline serology or antigen testing [18, 20, 24]. The final trial used stored participant blood samples, taken before 2020, as negative controls for subsequent serology analyses [26]. There were no relevant subgroup analyses, but a sensitivity analysis including seropositive BRACE trial participants showed concordant results [23].

#### Sample Size, Effect Size and Power

The power of a trial to detect the effect of an intervention is influenced by the event rate, the intervention's effect size and the sample size. The unknown trajectory of the COVID-19 pandemic made accurate predictions difficult during trial design.

**Figure 3.**
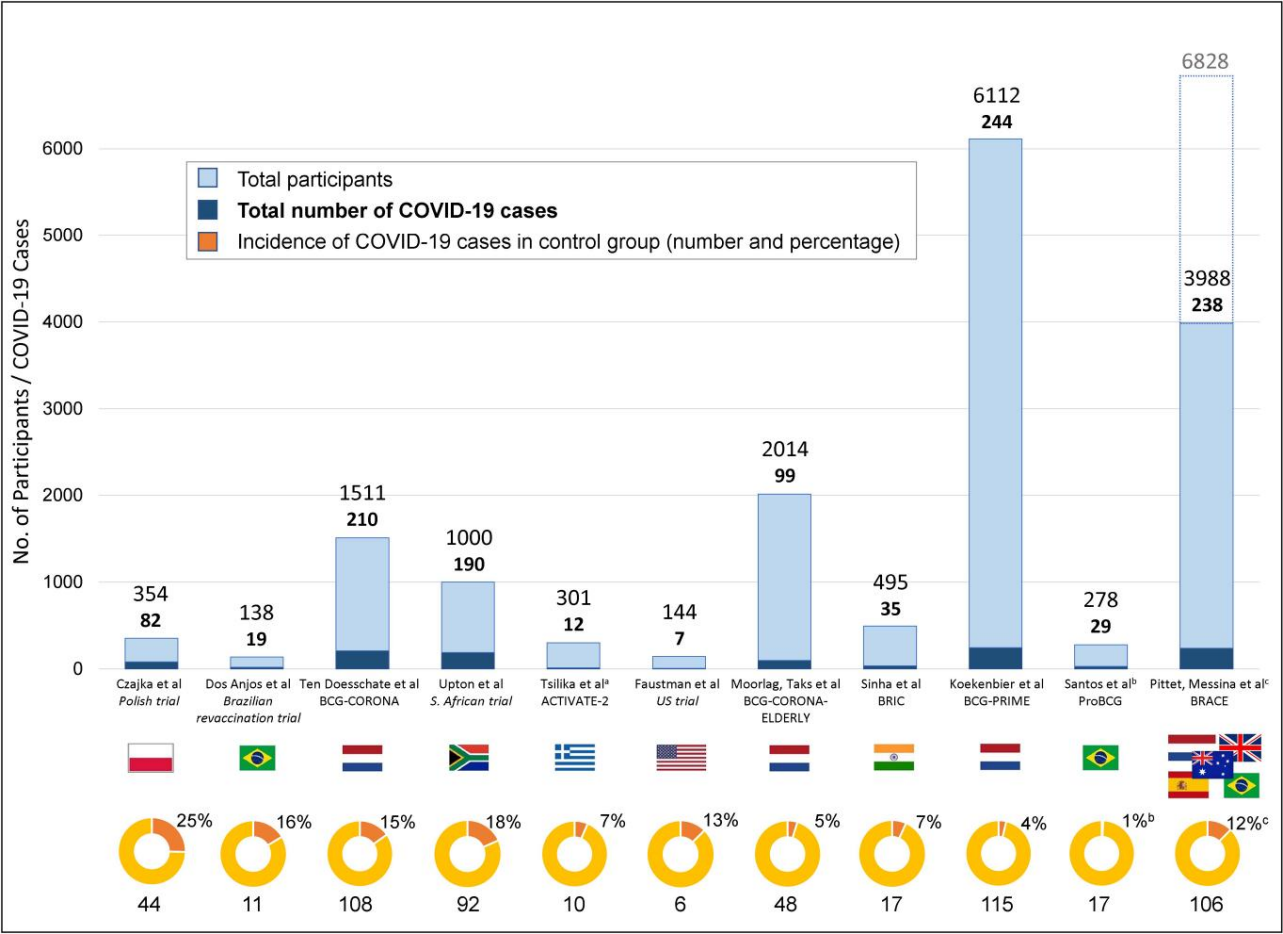
Incidence of coronavirus disease 2019 (COVID-19) in the trials of BCG vaccination for protection against COVID-19 [16–26]. Bar chart presents the total number of participants enrolled in each trial, along with the total number of reported COVID-19 cases (as defined by each trial). Pie chart presents data from the control group: the percentage of COVID-19 cases in the control group with the absolute number below. ^a^In the ACTIVATE-2 trial, the rates of COVID-19 at 3 months are presented, as per the primary outcome. ^b^In the ProBCG trial, the incidence percentage in the control group is as reported by the trial (using person-days of observation). ^c^In the BRACE trial, the percentage denotes the adjusted estimated probability of symptomatic COVID-19 in the control group at 6 months, as per the primary outcome (adjusted for stratification factors used at randomization: age, geographic location, and comorbid conditions); the extended bar and extra number include the participants recruited to stage 1 of the BRACE trial, who were not included in the primary outcome results.

The trials were done in different countries over different time periods and participants’ risk of infection with SARS-CoV-2 varied with the local incidence, prevalent variant and availability of PPE. The event rate was commonly overestimated in trial sample size calculations, where these were provided. There were between 7 and 244 total COVID-19 cases (with variable definition) in the trials, with an incidence between 4% and 25% in the control groups (Figure 3).

In the trials that provided a sample size calculation, the anticipated effect size of BCG vaccination varied from an 18% [23] to 50% reduction [17, 24] in COVID-19. Estimates for other outcomes ranged from a 20% reduction in workplace absence [20] to a 75% reduction in COVID-19 hospitalization [22].

Actual sample size varied between 138 and 6112 participants. In addition to overestimates in sample size calculations, several trials did not meet their recruitment targets because of the earlier-than-expected availability of COVID-19–specific vaccines, reducing their power and increasing the risk of type 2 error [16, 17, 19, 21, 23, 25].

### Intervention

#### BCG Strain and Dosing

The immune response to different BCG strains varies, although the impact of this on clinical protection against tuberculosis or off-target outcomes is debated [47–51]. Most trials reporting reductions in all-cause infant mortality rate have used BCG-Denmark [9]. Four different strains were used in the BCG COVID-19 trials (Table 1 and Supplementary Table 1*A*): BCG-Denmark in 5 trials [18, 20, 22–24], BCG-Russia in 3 [17, 19, 21], BCG-Moreau in 1 [16], and BCG-Japan in 1 [26]. One trial used both BCG-Russia and BCG-Moreau [25]. Ten trials used a single standard adult dose of BCG vaccine. The 11th trial used participants in an existing trial, with participants receiving ≥3 standard doses of BCG over a 2-year period [26]. This trial, which reported strong evidence of protection against COVID-19 despite its small size, was unique in a number of ways but notably was the only trial to use the BCG-Japan strain (which has a higher colony-forming unit count and higher immunogenicity than other strains [47, 52, 53]).

#### Other Vaccines

As well as their own off-target effects [54, 55], other vaccines modify the off-target effects of BCG vaccination in both infants and adults [8, 56–58]. Three trials reported vaccines received by participants before enrollment [18, 20, 24], and 2 reported those received during follow-up [23, 25]. Supplementary analyses in 2 trials with censoring at the time of any additional vaccination produced results concordant with the primary analysis [22, 23].

The introduction of COVID-19–specific vaccines affected most of the BCG COVID-19 trials (Supplementary Table 1*B*). While off-target effects or interactions with BCG are possible, the greatest impact was on recruitment and the reduction in SARS-CoV-2 infections during follow-up. In 6 trials, 29%–100% of participants received ≥1 dose, with no censoring of their follow-up in the primary analysis [17, 18, 20, 22, 24, 25]. Three trials completed follow-up before COVID-19–specific vaccinations were given [16, 26] or censored follow-up at the time of first vaccination for primary analysis [23]. Two trials did not report COVID-19–specific vaccination rates during follow-up [19, 21]. Results from sensitivity analyses of BCG-PRIME (follow-up censored at first COVID-19 vaccination) and the BRACE trial (follow-up extended beyond COVID-19 vaccination) were both concordant with the primary analyses [23, 24].

### Control

Complete blinding is an inevitable challenge in all BCG trials due to predictable injection site reactions and scarring. Presumption of randomization group by participants (or trial staff) based on presence or absence of scar might bias outcome assessment by influencing symptom reporting or testing for COVID-19. It might also influence exposure to SARS-CoV-2 by changing other behaviors, such as adherence to mask wearing and other personal protective measures. Ten trials were double-blinded and used saline placebo vaccinations (Table 1 and Supplementary Table 1*A*). One trial was not blinded to participants and delivered no intervention to the control group [17].

### Outcome

#### Choice of Outcome Measure

The choice of primary outcome and COVID-19 case definition (symptoms, and timing, type and availability of testing) differed between trials. Eight trials investigated the incidence of COVID-19 as (one of) their primary outcome(s) (Table 1 and Supplementary Table 1*B*). Three required symptoms in addition to a positive test [23, 24, 26], and 3 required participants to be tested when symptomatic but without symptoms as part of their case definition [16, 17, 25]. Another trial presented their primary outcome as 2 separate measures: “probable” (symptoms only) and “confirmed” (positive cartridge-based nucleic acid amplification test result) COVID-19 [19]. The eighth trial combined “possible” (symptoms only), “probable” (symptoms plus hospitalization/antibiotic prescription) and “definitive” (molecularly confirmed) COVID-19 [21]. The remaining 3 trials reported hospitalization with COVID-19 [22] or surrogate outcomes: absenteeism due to any illness [20] and respiratory tract infections requiring medical attention [18] as primary outcomes.

The BRACE trial had a coprimary outcome of severe COVID-19, which combined hospitalized and nonhospitalized severe disease (confined to bed or unable to work for ≥3 consecutive days) [23]. The US trial reported measures of infectious disease symptoms and severity as a second primary outcome [26].

The method and intensity of follow-up varied between trials, potentially affecting the case detection rate and risk of recall bias. Follow-up was daily or weekly in 4 trials [16, 18, 20, 23], initially weekly and then biweekly or monthly in 2 trials [24, 25], and every 1–3 months in the other 5 trials [17, 19, 21, 22, 26].

Some trials further assessed antibody responses to SARS-CoV-2 and COVID-19 vaccines in exploratory outcomes. In BCG-CORONA, more BCG-vaccinated participants seroconverted at 3 months compared with placebo-vaccinated, but this effect did not persist at 6 or 12 months [59]. Secondary analyses of 2 trials revealed no effect of BCG vaccination on subsequent serological responses to COVID-19 vaccinations [60, 61].

#### Measurement of Severe COVID-19

**Figure 4.**
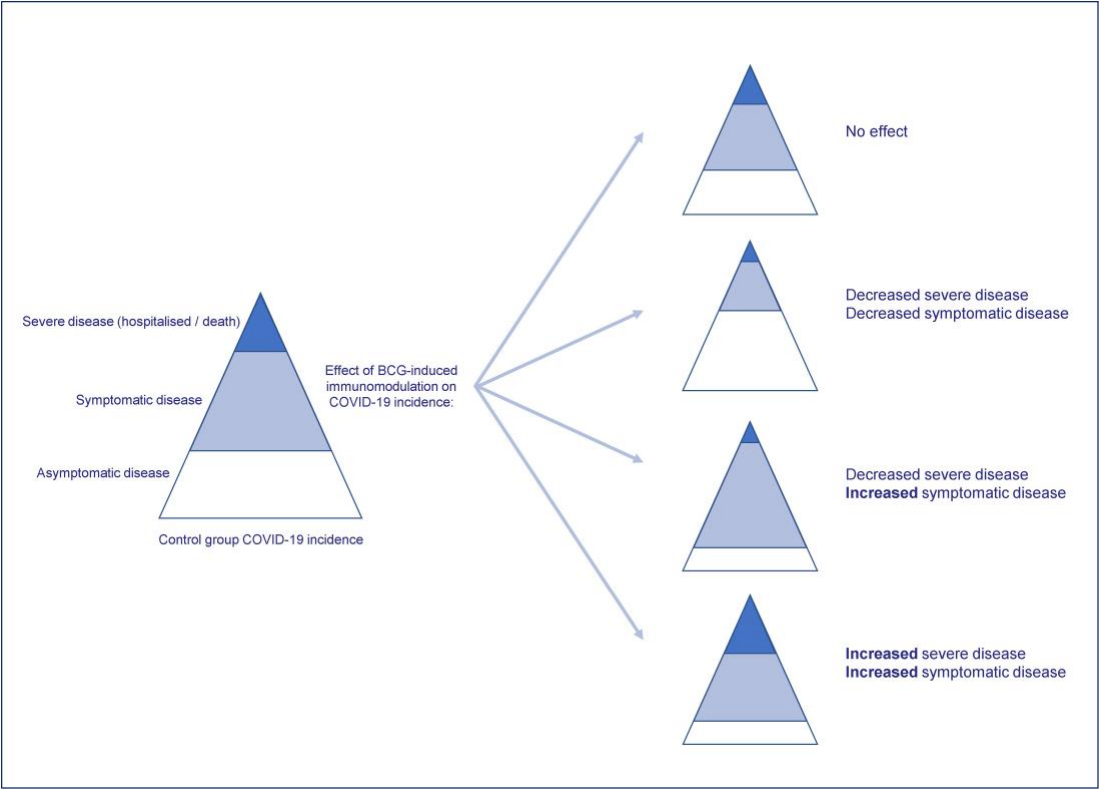
Potential effects of BCG vaccination on coronavirus disease 2019 (COVID-19) incidence. Four scenarios illustrate the potential effects of BCG vaccination on the incidence of symptomatic and severe COVID-19.

The beneficial off-target effects of BCG vaccination are proposed to result, at least in part, from trained immunity leading to an enhanced immune response: BCG vaccination increases proinflammatory cytokine production following heterologous stimulation in vitro in adults [32, 33]. However, in a subanalysis of BRACE trial participants, BCG vaccination modulated cytokine production and T-cell phenotypes following in vitro stimulation of whole blood with SARS-CoV-2, suggesting that BCG might suppress some of the pathological cytokine responses seen in severe COVID-19 [62]. It is possible that BCG vaccination enhances immune responses to SARS-CoV-2 that lead to more symptomatic disease while reducing those associated with severe disease (hospitalization and deaths) by inducing more effective viral clearance and reducing cytokine storm (Figure 4).

Two trials reported severe COVID-19 as a primary outcome [22, 23], although the BRACE trial definition included those with significant symptoms not requiring hospitalization. Most trials reported hospitalizations and deaths as a secondary outcome and were underpowered for this outcome.

### Timing

The follow-up duration of the BCG COVID-19 trials ranged between 3 and 15 months from randomization, and between 3 months and approximately 3 years from administration of initial BCG doses (Table 1 and Supplementary Table 1*B*). The US trial was an outlier, following up participants for the longest period after initiation of the parallel study, and notably, commencing follow-up at least 2 years after the first dose of BCG in the original trial [26]. In addition to the other differences in design, the longer interval between BCG vaccination and exposure might be relevant to the beneficial findings in the US trial.

### Other Factors

#### Responders

It might be possible to induce BCG off-target effects only in certain individuals or “responders.” In a study using yellow fever vaccine as an experimental viral infection after BCG vaccination, those with suppressed viremia (BCG responders) had epigenetic differences both before and 28 days after BCG vaccination, compared with “nonresponders” with higher viremia [32].

#### Risk of Bias

Finally, bias may have affected the results of the trials (see Supplementary Discussion and Supplementary Table 3).

## CONCLUSIONS

The findings of the 11 trials that have reported the effect of BCG vaccination on protection against COVID-19 are inconsistent. However, 3 of the largest trials suggest that BCG vaccination might increase the risk of COVID-19. Only the US trial of repeated BCG vaccination in diabetics reported strong evidence for a protective effect of BCG. Heterologous study design, particularly in relation to participant characteristics, outcome definitions and duration of follow-up, limits the potential for meta-analysis. The early availability of COVID-19 vaccines had a significant impact, preventing several trials reaching their intended sample size and reducing the number of COVID-19 episodes. This particularly limited the trials’ capacity to assess the effect on severe COVID-19, the outcome of most relevance.

Animal models of BCG vaccination against SARS-CoV-2 infection have also produced mixed results, with 1 study reporting protection against influenza A but not SARS-CoV-2 infection [63–65]. The relevance of these findings to humans is limited by differences in the route of BCG administration and severity of SARS-CoV-2 infection.

This review highlights several aspects of study design that influence the results of trials assessing the off-target effects of vaccines, including differences in participant demographics, intervention protocols, outcome measures, and timing of assessment. Although there was no clear single variable that explained the mixed results in the BCG COVID-19 trials, key factors included participant age and sex, unique characteristics of SARS-CoV-2 and the number of BCG doses. A number of previous BCG vaccination trials have shown clear beneficial effects, particularly in neonates in settings with high-mortality rates. Identifying the factors that explain the inconsistencies in results is challenging but key to exploiting the off-target effects of vaccines.

## Supplementary Data

Supplementary materials are available at *The Journal of Infectious Diseases* online. Consisting of data provided by the authors to benefit the reader, the posted materials are not copyedited and are the sole responsibility of the authors, so questions or comments should be addressed to the corresponding author.

## Supplementary Material

jiad316_Supplementary_Data

## Notes

***Author contributions.*** C. C. A. N. completed the literature search and, together with N. C., wrote the first draft of the manuscript. N. L. M. and L. F. P. revised the manuscript for critical content.

***Financial support*.** This work was supported by the European Society for Paediatric Infectious Diseases (fellowship award to C. C. A. N.), the Swiss National Science Foundation (early postdoctoral mobility grant P2GEP3_178155 to L. F. P.), and the National Health and Medical Research Council (investigator grant GNT1197117 to N. C.). Funding to pay the Open Access publication charges for this article was provided by The University of Melbourne.
