# Supplementary material for: Interpreting the Results of Trials of BCG Vaccination for Protection Against COVID-19
Source: J Infect Dis. 2023 Aug 10;228(10):1467–78. doi: 10.1093/infdis/jiad316 (PMC10640778; doi:10.1093/infdis/jiad316)
Supplement: jiad316_Supplementary_Data [file jiad316_supplementary_data.zip › SupplementaryDiscussion.docx]

**Supplementary Discussion**

**Effect of participant sex**

The effect of sex was reported in subgroup analysis by three trials, none of which reported strong evidence for a difference between males and females in the effect of BCG on their primary outcome [1-3]. However, there was some suggestion in all three trials that BCG was of more benefit (or less detriment) in females. In both the Brazilian revaccination trial and BCG-CORONA-ELDERLY, there was a higher incidence of events in the BCG group compared with the control group amongst males, with the reverse seen in females, although numbers were small [1, 2]. In the BRACE trial, the disease-free survival curves for BCG and placebo groups separated earlier in males compared to females (with more COVID-19 cases in the BCG group in both) [3].

**Risk of bias**

Bias may have affected the results of the trials (Supplementary Table 3). Ten trials were judged to have ‘some concerns’ in at least one domain [1-10]. Given the predictable skin reaction following BCG vaccination, participants were potentially unblinded and participant-reported outcomes, or those affected by participant behaviour, susceptible to bias. In several trials, there was insufficient information on analysis methods and/or participant exclusions post-randomisation [1, 2, 4, 5, 10]. In seven trials, analysis plans for the final primary outcome were not publicly pre-specified before publication [2, 5], were not specified in sufficient detail [1, 6, 7, 10] or were changed at an unknown time from those initially specified at registration [4], meaning there was potential for bias in selection of the results reported.

**References**

1. Dos Anjos LRB, da Costa AC, Cardoso A, et al. Efficacy and Safety of BCG Revaccination With M. bovis BCG Moscow to Prevent COVID-19 Infection in Health Care Workers: A Randomized Phase II Clinical Trial. Front Immunol **2022**; 13:841868.

2. Moorlag S, Taks E, Ten Doesschate T, et al. Efficacy of BCG Vaccination Against Respiratory Tract Infections in Older Adults During the Coronavirus Disease 2019 Pandemic. Clin Infect Dis **2022**; 75:e938-e46.

3. Pittet LF, Messina NL, Orsini F, et al. Randomized Trial of BCG Vaccine to Protect against Covid-19 in Health Care Workers. N Engl J Med **2023**; 388:1582-96.

4. Czajka H, Zapolnik P, Krzych Ł, et al. A Multi-Center, Randomised, Double-Blind, Placebo-Controlled Phase III Clinical Trial Evaluating the Impact of BCG Re-Vaccination on the Incidence and Severity of SARS-CoV-2 Infections among Symptomatic Healthcare Professionals during the COVID-19 Pandemic in Poland-First Results. Vaccines (Basel) **2022**; 10.

5. Faustman DL, Lee A, Hostetter ER, et al. Multiple BCG vaccinations for the prevention of COVID-19 and other infectious diseases in type 1 diabetes. Cell Rep Med **2022**; 3:100728.

6. Sinha S, Ajayababu A, Thukral H, et al. Efficacy of Bacillus Calmette-Guérin (BCG) Vaccination in Reducing the Incidence and Severity of COVID-19 in High-Risk Population (BRIC): a Phase III, Multi-centre, Quadruple-Blind Randomised Control Trial. Infect Dis Ther **2022**:1-13.

7. Tsilika M, Taks E, Dolianitis K, et al. ACTIVATE-2: A Double-Blind Randomized Trial of BCG Vaccination Against COVID-19 in Individuals at Risk. Front Immunol **2022**; 13:873067.

8. Ten Doesschate T, van der Vaart TW, Debisarun PA, et al. Bacillus Calmette-Guérin vaccine to reduce healthcare worker absenteeism in COVID-19 pandemic, a randomized controlled trial. Clin Microbiol Infect **2022**; 28:1278-85.

9. Koekenbier EL, Fohse K, van de Maat JS, et al. Bacillus Calmette-Guérin vaccine for prevention of COVID-19 and other respiratory tract infections in older adults with comorbidities: a randomized controlled trial. Clin Microbiol Infect **2023**.

10. Santos AP, Werneck GL, Dalvi APR, et al. The effect of BCG vaccination on infection and antibody levels against SARS-CoV-2-The results of ProBCG: a multicenter randomized clinical trial in Brazil. Int J Infect Dis **2023**; 130:8-16.
