# Supplementary material for: Interpreting the Results of Trials of BCG Vaccination for Protection Against COVID-19
Source: J Infect Dis. 2023 Aug 10;228(10):1467–78. doi: 10.1093/infdis/jiad316 (PMC10640778; doi:10.1093/infdis/jiad316)
Supplement: jiad316_Supplementary_Data [file jiad316_supplementary_data.zip › SupplementaryFigure1.docx]

**Identification of studies via databases and registers**

Records identified from:

PubMed (n = 497)

Clinicaltrials.gov registry (n = 34)

WHO trials registry (n = 55)

Records removed before screening:

Duplicate records removed

(n = 5)

**Identification**

Records excluded after title and abstract screening, including trial registry entries after searching for published results

(n = 520)

Records screened

(n = 581)

Reports sought for full text review

(n = 61)

**Screening**

Reports excluded (n=51):

Protocol (n = 4)

Report non-primary outcomes (n = 5)

Review, comment or letter with no new relevant data (n=35)

Inclusion criteria not met (n=7)

Reports assessed for eligibility

(n = 61)

Studies included in review

**(n = 10**

**(+ 1 in press))**

**Included**

**Supplementary Figure 1 – PRISMA 2020 flow diagram for literature search.**

A literature search containing the terms ‘BCG’ AND (‘COVID’ OR ‘SARS-CoV-2’) was last run on 9/3/23. In addition, searches were made of clinicaltrials.gov (https://clinicaltrials.gov, last search on 5/1/23) and World Health Organization International Clinical Trials Registry Platform (https://trialsearch.who.int, last search on 6/1/23) to identify planned trials. The BRACE trial was in press and also included in the review. *From:*  Page MJ, McKenzie JE, Bossuyt PM, Boutron I, Hoffmann TC, Mulrow CD, et al. The PRISMA 2020 statement: an updated guideline for reporting systematic reviews. BMJ 2021;372:n71. doi: 10.1136/bmj.n71. For more information, visit: <http://www.prisma-statement.org/>.
