# Supplementary material for: Interpreting the Results of Trials of BCG Vaccination for Protection Against COVID-19
Source: J Infect Dis. 2023 Aug 10;228(10):1467–78. doi: 10.1093/infdis/jiad316 (PMC10640778; doi:10.1093/infdis/jiad316)
Supplement: jiad316_Supplementary_Data [file jiad316_supplementary_data.zip › SupplementaryMethods.docx]

**Supplementary Methods**

**Details of literature search**

A literature search containing the terms ‘BCG’ and (‘COVID’ or ‘SARS-CoV-2’), and last run on 9^th^ March 2023, identified 497 articles, of which 10 met inclusion criteria. One further trial was in press [1] (Table 1, Figure 2, Supplementary Tables 1 and 2, Supplementary Figure 1). Two trial registries identified a significant number of trials which remain unpublished (Supplementary Table 4). Of the 11 published trials, 10 were designed specifically to investigate the effect of BCG on COVID-19 [1-10], while one was an amendment to an existing trial investigating the effect of BCG on type 1 diabetes mellitus (T1DM) [11]. One further trial investigating the effect of VPM1002 (a modified form of BCG) on severe respiratory disease, including COVID-19, in elderly participants was identified but not included in the analysis [12].

**References**

1. Pittet LF, Messina NL, Orsini F, et al. Randomized Trial of BCG Vaccine to Protect against Covid-19 in Health Care Workers. N Engl J Med **2023**; 388:1582-96.

2. Czajka H, Zapolnik P, Krzych Ł, et al. A Multi-Center, Randomised, Double-Blind, Placebo-Controlled Phase III Clinical Trial Evaluating the Impact of BCG Re-Vaccination on the Incidence and Severity of SARS-CoV-2 Infections among Symptomatic Healthcare Professionals during the COVID-19 Pandemic in Poland-First Results. Vaccines (Basel) **2022**; 10.

3. Dos Anjos LRB, da Costa AC, Cardoso A, et al. Efficacy and Safety of BCG Revaccination With M. bovis BCG Moscow to Prevent COVID-19 Infection in Health Care Workers: A Randomized Phase II Clinical Trial. Front Immunol **2022**; 13:841868.

4. Moorlag S, Taks E, Ten Doesschate T, et al. Efficacy of BCG Vaccination Against Respiratory Tract Infections in Older Adults During the Coronavirus Disease 2019 Pandemic. Clin Infect Dis **2022**; 75:e938-e46.

5. Sinha S, Ajayababu A, Thukral H, et al. Efficacy of Bacillus Calmette-Guérin (BCG) Vaccination in Reducing the Incidence and Severity of COVID-19 in High-Risk Population (BRIC): a Phase III, Multi-centre, Quadruple-Blind Randomised Control Trial. Infect Dis Ther **2022**:1-13.

6. Ten Doesschate T, van der Vaart TW, Debisarun PA, et al. Bacillus Calmette-Guérin vaccine to reduce healthcare worker absenteeism in COVID-19 pandemic, a randomized controlled trial. Clin Microbiol Infect **2022**; 28:1278-85.

7. Tsilika M, Taks E, Dolianitis K, et al. ACTIVATE-2: A Double-Blind Randomized Trial of BCG Vaccination Against COVID-19 in Individuals at Risk. Front Immunol **2022**; 13:873067.

8. Upton CM, van Wijk RC, Mockeliunas L, et al. Safety and efficacy of BCG re-vaccination in relation to COVID-19 morbidity in healthcare workers: A double-blind, randomised, controlled, phase 3 trial. EClinicalMedicine **2022**; 48:101414.

9. Koekenbier EL, Fohse K, van de Maat JS, et al. Bacillus Calmette-Guérin vaccine for prevention of COVID-19 and other respiratory tract infections in older adults with comorbidities: a randomized controlled trial. Clin Microbiol Infect **2023**.

10. Santos AP, Werneck GL, Dalvi APR, et al. The effect of BCG vaccination on infection and antibody levels against SARS-CoV-2-The results of ProBCG: a multicenter randomized clinical trial in Brazil. Int J Infect Dis **2023**; 130:8-16.

11. Faustman DL, Lee A, Hostetter ER, et al. Multiple BCG vaccinations for the prevention of COVID-19 and other infectious diseases in type 1 diabetes. Cell Rep Med **2022**; 3:100728.

12. Blossey AM, Brückner S, May M, et al. VPM1002 as Prophylaxis Against Severe Respiratory Tract Infections Including Coronavirus Disease 2019 in the Elderly: A Phase 3 Randomized, Double-Blind, Placebo-Controlled, Multicenter Clinical Study. Clin Infect Dis **2023**; 76:1304-10.
