# Supplementary material for: Interpreting the Results of Trials of BCG Vaccination for Protection Against COVID-19
Source: J Infect Dis. 2023 Aug 10;228(10):1467–78. doi: 10.1093/infdis/jiad316 (PMC10640778; doi:10.1093/infdis/jiad316)
Supplement: jiad316_Supplementary_Data [file jiad316_supplementary_data.zip › SupplementaryTable1a.docx]

|  | **REGISTRATION** | | **PARTICIPANTS** | | | | | **INTERVENTION** | | **CONTROL** |
| --- | --- | --- | --- | --- | --- | --- | --- | --- | --- | --- |
| First author, year,  (trial name);  location, trial dates | Type of study, registration (clinicaltrials.gov if not stated), date registered | Publication of protocol and SAP | Enrolment criteria; age, sex | Previous BCG, pre-existing BCG scar | TST or IGRA testing at baseline | SARS-CoV-2 serology or test at baseline | Total number enrolled (planned enrolment) | BCG strain and dosing | Randomisation (ratio), blinding | Control group |
| **Czajka, 22;** Poland, Jul 20 - Mar 21 | RCT, NCT04648800, registered in Nov 20 (plus EudraCT 2020-002111-22) | Not found | Healthcare workers >25 years; mean age 46y, 81% female | All presumed to have received >2 previous BCG doses, some had multiple scars but overall scar prevalence not reported | TST at baseline, excluded from randomisation if diameter >5mm (50.6% of those screened) | Serology testing at baseline, excluded if positive | 717 enrolled, of whom 354 were randomised, plus 363 with positive TST, not randomised & analysed separately  (target 1000 total) | BCG (-10, BIOMED-Lublin SA (Moreau strain)), single dose after randomisation | Randomised (1:1), double-blind | Placebo (saline), single dose after randomisation |
| **Dos Anjos, 22;** Brazil, Aug 20 - Aug 21 | RCT, RBR-4kjqtg (REBEC, Brazilian registry), registered in May 20 | Protocol published in Oct 20^1^, SAP not found | Healthcare workers with previous BCG (and scar); mean age 43y, 76% female | All reported previous BCG, 86.3% had an existing scar | Not described | Serology testing at baseline, excluded if positive | 138 enrolled (target 394) | BCG (Moscow strain), single dose after randomisation | Randomised (1:1), participants not blinded, lab staff and outcome assessors blinded | No intervention |
| **ten Doesschate, 22,**  **BCG-CORONA;** Netherlands, Mar 20 - Mar 21 | RCT, NCT04328441, registered in Mar 20 (plus EudraCT 2020-000919-69) | Protocol published in Jun 20^2^, SAP uploaded to clinicaltrials.gov in May 20 | Healthcare workers; mean age 42y, 74% female | 16.9% reported prior BCG, scar prevalence not reported | Not described | Not described. 2 participants had previously tested positive for COVID-19 | 1511 enrolled (target 1500) | BCG (Danish strain 1331, SSI), single dose after randomisation | Randomised (1:1), double-blind | Placebo (saline), single dose after randomisation |
| **Upton, 22;** South Africa, May 20 - Oct 21 | RCT, NCT04379336, registered in May 20 | Protocol included in supplementary materials, no pre-publication found | Healthcare workers; median age 39y, 70% female | Presumed that all participants had prior BCG in childhood, 49.6% had an existing scar | IGRA testing at baseline (48.5% positive) and 52w (47.2% positive), included in analysis | Serology testing at baseline, 15.3% tested positive and were included in analysis | 1000 enrolled (target 500) | BCG (Danish strain 1331, SSI), single dose after randomisation | Randomised (1:1), double-blind | Placebo (saline), single dose after randomisation |
| **Tsilika, 22,**  **ACTIVATE-2;** Greece, Jun 20 - Apr 21 | RCT, NCT04414267, registered in Jun 20 (plus EudraCT 2020-002448-21) | Not found | Adults >50y with significant comorbidity (CAD/ COPD/ Charlson's comorbidity index >3); mean age 69y, 32% female | Unknown (national policy was for BCG at birth during relevant period), scar prevalence not reported | TST at baseline, excluded if diameter >10mm (40.3% of those screened) | Rapid serology testing at baseline, excluded if positive | 301 enrolled (target 900) | BCG (Moscow strain 361-I), single dose after randomisation | Randomised (1:1), double-blind | Placebo (saline), single dose after randomisation |
| **Faustman, 22;** USA, Jan 20 - Apr 21 | Amendment to an existing RCT, NCT02081326 (original trial), updated with details of parallel study in Jul 21 | Not found | Adults 18-50y, with type 1 diabetes mellitus; median age 44y, 42% female | No, an exclusion criterion | Not stated whether this was done at recruitment to original trial | All had negative serology before the parallel trial commenced. These samples were used as comparison for serology results taken during follow up | 144 (of 150 original participants) enrolled in this parallel study (target unknown) | BCG-Japan (Tokyo-172 strain), 3 doses given prior to Jan 20 (2 doses given 4 weeks apart then an annual booster dose). Not stated whether further doses were given during this parallel trial | Randomised (2 BCG: 1 placebo), double-blind | Placebo (saline), doses given as per BCG group |
| **Moorlag & Taks, 22, BCG-CORONA-ELDERLY;** Netherlands, Apr 20 - May 21 | RCT, NCT04417335, registered in Jun 20 (plus EudraCT 2020-001591-15) | Protocol published in Jun 20^2^ (different primary outcome planned), SAP not found | Adults >60y; median age 67y, 47% female | 27.3% with previous BCG (18.9% unknown), scar prevalence not reported | Not described | No testing at baseline, no participants had documented COVID-19 before randomisation | 2014 enrolled (target 2000) | BCG (Danish strain 1331, SSI), single dose after randomisation | Randomised (1:1), double-blind | Placebo (saline), single dose after randomisation |
| **Sinha, 22,**  **BRIC;**  India, Oct 20 - Dec 21 | RCT, CTRI/2020/ 07/026668 (Clinical Trials Registry India), registered in Jul 20 | Not found | Adults 18-60y with significant comorbidity (diabetes, CKD, lung disease, CV disease); mean age 44y, 48% female | Unknown (BCG is in national immunisation programme, about 70% coverage), scar prevalence not reported | Not described | Serology testing and antigen test at baseline, excluded if either was positive | 495 enrolled (target 800) | BCG (Moscow strain, SII), single dose after randomisation | Randomised (1:1), double-blind | Placebo (saline), single dose after randomisation |
| **Koekenbier, 23; BCG-PRIME;** Netherlands, Sep 20 – Jul 21 | RCT, NCT04537663, registered in Sep 20 (plus EUCTR2020-  003470-47-NL) | Protocol not found, SAP uploaded to clinicaltrials.gov in Jan 21 | Adults >60y with recent hospitalisation or comorbidity; median age 69y, 37% female | 14.9% previous BCG (9.5% unknown), scar prevalence not reported | Not described | No testing at baseline, no participants had documented COVID-19 before randomisation | 6112 enrolled (target 5200-7000) | BCG (Danish strain 1331, SSI), single dose after randomisation | Randomised (1:1), double-blind | Placebo (saline), single dose after randomisation |
| **Santos, 23; ProBCG;**  Brazil, Oct 20 – May 22 | RCT, NCT04659941, registered in Dec 20 (plus RBR-5ysj54, Jul 20) | Protocol and SAP uploaded to clinicaltrials.gov in Sep 21 | Healthcare workers; median/ mean age not given, 80% female | 93.2% had >1 previous BCG, scar prevalence not reported | IGRA testing at baseline (13.4% positive), included in analysis | Serology testing and swab at baseline, excluded if either was positive (serology results disregarded if prior COVID-19 vaccination) | 278 enrolled (target 752) | BCG (23% received Moreau strain, 77% Moscow strain, SII), single dose after randomisation | Randomised (1:1), double-blind | Placebo (saline), single dose after randomisation |
| **Pittet & Messina, 23,**  **BRACE;** Australia, Brazil, Netherlands, Spain, UK, May 20 - May 22 | RCT, NCT04327206, registered in Mar 20 | Protocol published in Oct 21^3^, SAP first uploaded to clinicaltrials.gov in Feb 21 | Healthcare workers; mean age 43y, 75% female | 76.7% with previous BCG, scar prevalence not reported | TST not done at baseline, IGRA testing done for Brazilian participants only (results not reported) | Serology testing at baseline (14.1% positive), plus PCR testing in Brazil (1.8% positive). Exclusion from primary analysis (mITT) if either was positive | 3988 enrolled in stage 2 (target 7244 in stage 2). Also 2840 participants enrolled in stage 1 but excluded from primary analysis. | BCG (Danish strain 1331, SSI), single dose after randomisation | Randomised (1:1), double-blind | Placebo (saline), single dose after randomisation |

**Supplementary Table 1a – Details of trial registration, pre-publication of protocol and SAP, randomisation and blinding, participants and baseline testing, intervention and control**

BCG – Bacillus Calmette–Guérin vaccination; CAD – coronary artery disease; CKD – chronic kidney disease; COPD – chronic obstructive pulmonary disease; CV disease – cardiovascular disease; IGRA – interferon gamma release assay test; mITT – modified intention to treat; PCR – polymerase chain reaction; RCT – randomised controlled trial; SAP – statistical analysis plan; TST – tuberculin skin test.

References:

^1^ Junqueira-Kipnis, A. P. *et al.* BCG revaccination of health workers in Brazil to improve innate immune responses against COVID-19: A structured summary of a study protocol for a randomised controlled trial. *Trials* **21**, 881, doi:10.1186/s13063-020-04822-0 (2020).

^2^ Ten Doesschate, T. *et al.* Two Randomized Controlled Trials of Bacillus Calmette-Guérin Vaccination to reduce absenteeism among health care workers and hospital admission by elderly persons during the COVID-19 pandemic: A structured summary of the study protocols for two randomised controlled trials. *Trials* **21**, 481, doi:10.1186/s13063-020-04389-w (2020).

^3^ Pittet, L. F. *et al.* BCG vaccination to reduce the impact of COVID-19 in healthcare workers: Protocol for a randomised controlled trial (BRACE trial). *BMJ Open* **11**, e052101, doi:10.1136/bmjopen-2021-052101 (2021).
