# Supplementary material for: Interpreting the Results of Trials of BCG Vaccination for Protection Against COVID-19
Source: J Infect Dis. 2023 Aug 10;228(10):1467–78. doi: 10.1093/infdis/jiad316 (PMC10640778; doi:10.1093/infdis/jiad316)
Supplement: jiad316_Supplementary_Data [file jiad316_supplementary_data.zip › SupplementaryTable1b.docx]

|  | **OUTCOME** | | | | | **TIMING/ FOLLOW UP** | | | |
| --- | --- | --- | --- | --- | --- | --- | --- | --- | --- |
| Reference (first author, year, (trial name), location) | Primary outcome(s) | Further detail of primary outcome definition(s) | Primary outcome results | Secondary outcomes published  (even if not pre-specified) |  | Follow up period; nature of active follow up | COVID-19 vaccination during trial follow up | Timing of COVID-19 serology testing; results during trial (see Supp Table 1a for baseline results) | Participants lost to follow up or excluded from analysis, any description of missing data |
| Czajka, 22, Poland | Occurrence of PCR-confirmed SARS-CoV-2 infections | PCR-confirmed, no requirement for symptoms | 38/168 cases (22.6%) in BCG group and 44/174 cases (25.3%) in placebo group (p=0.7 for difference between groups, including non-randomised group) | COVID-19 in participants with pre-existing BCG scars and in healthcare worker groups | | 3 months; weekly phone contact | Appeared to complete follow up for primary analysis before any participant received COVID-19 vaccination* | Baseline, 6-8w, 3m; 23.1% seroconverted | 9 (5.1%) excluded or lost to follow up in BCG group, 3 (1.7%) in placebo group. Missing data not described |
| Dos Anjos, 22, Brazil | Cumulative incidence of COVID-19 cases | Confirmed by serology or molecular test (PCR or IgM/ IgG serology), no requirement for symptoms | 8/64 cases (12.5%) in BCG group, 11/67 (16.4%) in control group (vaccine efficacy 30% (95% CI -78 to 72%)) | COVID-19 cases with censoring for COVID-19 vaccination, symptomatic COVID-19, COVID-19 if pre-existing BCG scar, COVID-19 by sex, COVID-19 symptoms, innate immune activation | | 180 days; video calls on day 7, 15, 30, 60, 180 | 88% received a COVID-19 vaccination and were included in primary analysis | Baseline, 15d, 180d (plus before/ up to 14 days after COVID-19 vaccination); serology results not reported | No loss to follow up, 4 (5.9%) excluded in BCG group, 3 (4.3%) in control group. Missing data not described |
| ten Doesschate, 22, BCG-CORONA, Netherlands | Number of days of unplanned absenteeism for any illness | Number of days of illness, including quarantine, as a proportion of planned work days | 2.8% in BCG group, 2.7% in placebo group (adjusted RR 0.94 (0.78-1.15)).  (26.3% of unplanned absent days were due to quarantine while not too sick to work) | Cumulative incidence of documented COVID-19, self-reported acute respiratory symptoms or fever, death/ ICU admission/ hospital admission/ days of unplanned absenteeism due to documented COVID-19. Post-hoc sub-group analyses by prior BCG/ positive TST and age | | Median follow up 358d; mobile phone app with daily surveys for 6 months, then weekly surveys | 29.1% received >1 dose of a COVID-19 vaccination and were included in primary analysis | 12w and at end of study; serology results not reported | 31 (4.1%) lost to follow up in BCG group, 52 (6.8%) in placebo group. Median of 2 follow up days missing in BCG group, 1 in placebo group (6.2% and 7.2% total follow up days missing respectively) |
| Upton, 22, South Africa | Hospitalisation due to COVID-19 | Minimum of overnight admission to a hospital facility associated with a positive SARS-CoV-2 PCR test | 10/500 (2%) in BCG group, 5/500 (1%) in placebo group (HR 2.0 (95% CI 0.69-5.9)) | Incidence of antigen-confirmed COVID-19 and RTI symptoms, SARS-CoV-2 seroconversion, incidence of hospitalisation (all cause), severity of COVID-19 and RTIs, deaths due to COVID-19/ any cause, prevalence of latent TB at 52w, incidence of active TB | | 52 weeks; phone/ text/ email follow up at least 4 weekly | 67.9% received a COVID-19 vaccination and were included in primary analysis | Baseline, 10w, 26w and 52w; 33.1% seroconverted | 20 (4.0%) lost to follow up in BCG group, 16 (3.2%) in placebo group. Missing data not described (but stated that imputation was not required) |
| Tsilika, 22, ACTIVATE-2, Greece | Total cases of possible/ probable/ definitive COVID-19 in first 3 months after randomisation | Possible – symptomatic (>2 of cough, SOB, fever, expectoration, all >48h); probable – symptomatic (as above) + hospitalised/ prescribed antibiotic; definitive – positive molecular test | 2/148 (1.4%) in BCG group, 10/153 (6.5%) in placebo group (p=0.086) at day 90±5 (breakdown of case type not given). | Total cases of possible/ probable/ definitive COVID-19 at 4.5m and 6m after randomisation, severe COVID-19 requiring hospitalisation, prevalence of SARS-CoV-2 IgG/ IgM at 3m | | 3/6 months; 1.5 monthly follow up in person/ by phone | Did not report whether any participants received COVID-19 vaccination during trial | Baseline, 3m (rapid tests); 3% positive at 3m | No loss to follow up at 3m (for primary outcome) but 56 (37.8%) lost after this timepoint in BCG group, 55 (35.9%) in placebo group. Missing data not described |
| Faustman, 22, USA | Original trial: change in glycaemic control (HbA1c values).  Parallel study: incidence of symptomatic and confirmed COVID-19, infectious disease symptoms and severity | COVID-19: >1 FDA-defined symptom plus positive result on >5 of 10 assays (1-8: antibody result >3 SD higher than pre-trial antibody levels on protein array serology assays; 9: positive ELISA for anti-RBD antibodies; 10: positive PCR, RAT or point of care antibody test).  Infectious disease: Total and Average ID Index scores | 1/96 cases (1.0%) in BCG group, 6/48 (12.5%) in placebo group (92% vaccine efficacy, *p*=0.006).  Total ID symptom index: 48 ± 11 [n = 31] in BCG group, 152 ± 70 [n = 20] in placebo group (p = 0.04). Average ID symptom index: 13 ± 2 [n = 31] in BCG group, 23 ± 7 [n = 20] in placebo group (p =0.04) | Average workdays missed, individual infection symptom severity, household members' total infectious symptom index | | 15 months; 2 monthly via email, 6 monthly clinic visits | No COVID-19 vaccinations received during follow up period | 6 monthly (9 different serology tests); results varied for each serology test | No loss to follow up, missing data not described. 6/150 original participants were not included in the parallel trial, reason not specified |
| Moorlag & Taks, 22, BCG-CORONA-ELDERLY, Netherlands | Cumulative incidence of clinically relevant RTIs  (changed from cumulative incidence of COVID-19-related hospitalisation) | Participant-reported symptoms (>1 resp + >1 systemic) requiring medical intervention (initiation of antibiotic/ antiviral/ steroids, change to maintenance medication or hospitalisation) within 5 days of onset | 2.9% in BCG group vs 2.4% in placebo group, SHR 1.26 (98.2% CI 0.65-2.44)) | Cumulative incidence of documented SARS-CoV-2 infection, self-reported RTIs (with/ without medical intervention), self-reported symptoms of infection, hospitalisation or death due to COVID-19. Post-hoc subgroup analyses by age, sex, previous BCG, comorbidities | | 12 months; mobile phone app with daily surveys for 6 months, then weekly surveys (paper surveys and monthly phone call if no smartphone) | 80.9% received >1 dose of a COVID-19 vaccination and were included in primary analysis | Serology testing only done for a subset of participants who had a positive PCR | 19 (1.9%) excluded or lost to follow up in BCG group, 37 (3.7%) in placebo group. 95.8% of all participants completed follow up, but missing data otherwise not reported |
| Sinha, 22, BRIC, India | Incidence of COVID-19 | Confirmation by CB-NAAT testing (definite COVID-19) or by symptom screening (probable COVID-19) | Definite: 18/246 (7.3%) in BCG group, 17/249 (6.8%) in placebo group (OR 1.08 (95% 0.54-2.14)).  Probable: 15/246 (6.1%) in BCG group, 36/249 (14.5%) in placebo group (OR 0.38 (95% CI 0.20-0.72)) | Incidence of severe COVID-19, hospitalisation, oxygen requirement, ICU stay, mortality, SARS-CoV-2 seroconversion | | 9 months; (?in-person) follow up at 1m, 3m, 6m, 9m | Did not report whether any participants received COVID-19 vaccination during trial | Baseline, 3m, 6m and 9m; 85.4% positive at 9m | 15 (6.1%) excluded or lost to follow up in BCG group, 17 (6.8%) in placebo group, missing data not reported |
| Koekenbier, 23, BCG-PRIME, Netherlands | Incidence of COVID-19 | Symptoms compatible with COVID-19 and infection confirmed with PCR, antigen test or CT scan | 129 events (cumulative incidence 0.042) in BCG group, 115 (cumulative incidence 0.038) in placebo group (SHR 1.12 (95% CI 0.87-1.44)) | Clinically relevant RTI, asymptomatic, mild/ moderate and severe SARS-CoV-2 infections, pneumonia, medically attended RTIs, RTI-related hospital admission, RTI-like symptoms, measures of mental/ physical/ social functioning, subgroup analysis by previous BCG | | 6 months; mobile phone app (or phone call) weekly for 4w then bi-weekly | 71% received >1 dose of a COVID-19 vaccination and were included in primary analysis | No serology testing | 41 (1.3%) excluded, died or lost to follow up in BCG group, 47 (1.5%) in placebo group. 98.9% of planned questionnaires were completed (96.5% participants completed all questionnaires) |
| Santos, 23, ProBCG,  Brazil | Incidence of COVID-19 | Confirmation by PCR or serology | 12 cases (0.52%) in BCG group, 17 (0.75%) in placebo group (HR 0.65 (95%CI 0.31-1.39)). Incidence uses person-days of observation | Time to COVID-19, symptoms of COVID-19, work absenteeism, SARS-CoV-2 antibody response (before and after COVID-19-specific vaccination) | | 6 months; weekly (first 3m) then monthly phone/ email survey, plus in-person follow up at 10d, 3m, 6m | All those included in primary analysis received >1 doses of a COVID-19 vaccination (25.4% received >1 dose before trial enrolment) | Baseline, 10d, 3m, 6m; seroconversion rate not reported (and not interpretable due to universal COVID-19 vaccination) | 6 (4.3%) excluded or lost to follow up in BCG group, 8 (5.8%) in placebo group, missing data not reported |
| Pittet & Messina, 23, BRACE, Australia, Brazil, Netherlands, Spain, UK | Incidence of symptomatic and severe COVID-19 in first 6m after randomisation | Symptomatic: fever or >1 respiratory symptom with positive PCR, RAT or serology.  Severe: as per symptomatic, plus death, hospitalisation or non-hospitalised severe disease (non-ambulant or unable to work for >3 consecutive days) as a consequence of COVID-19 | 132/1703 (adj estimated probability 14.7%) symptomatic cases in BCG group, 106/1683 (12.3%) in placebo group (adj difference +2.4% (95% CI -0.7 - 5.5%)). 75/1703 (adj estimated probability 7.6%) severe cases in BCG group, 61/1683 (6.5%) in placebo group (adj difference +1.1% (95% CI -1.2 - 3.5%)) | Time to COVID-19, complications (pneumonia, oxygen requirement, hospitalisation, ICU admission, mechanical ventilation, death) due to COVID-19, days of unplanned absenteeism, asymptomatic COVID-19, number of days with symptoms, subgroup analyses by age, sex, location, comorbidities, previous BCG | | 6/ 12 months; weekly follow up via smartphone app/ phone call/ text message (daily if unwell) | Follow up censored at time of first COVID-19 vaccination for primary analysis | Baseline, 3m, 6m, 9m and 12m; serology results not reported after baseline | 35 (1.8%) lost to follow up in BCG group, 35 (1.8%) in placebo group. For primary analysis, follow up was censored if symptom data was missing for >3 days or there was no test result for an illness episode (mITT population, including 3386/3988 participants (84.9%)) |

**Supplementary Table 1b – Details of trial follow up and outcomes**

* unable to confirm with authors.

Adj – adjusted; BCG – Bacillus Calmette–Guérin vaccination; CB-NAAT - cartridge- based nucleic acid amplification test; CI – confidence interval; ELISA - enzyme-linked immunosorbent assay; FDA – US Food and Drug Administration; ICU – intensive care unit; ID – infectious disease; IGRA – interferon gamma release assay test; m – months; mITT – modified intention to treat; OR – odds ratio; PCR – polymerase chain reaction; RAT – rapid antigen test; RBD – receptor binding domain; resp – respiratory; RR – risk ratio; RTI – respiratory tract infection; SD – standard deviation; SHR – sub-distribution hazard ratio; SOB – shortness of breath; TST – tuberculin skin test.
