# Supplementary material for: Interpreting the Results of Trials of BCG Vaccination for Protection Against COVID-19
Source: J Infect Dis. 2023 Aug 10;228(10):1467–78. doi: 10.1093/infdis/jiad316 (PMC10640778; doi:10.1093/infdis/jiad316)
Supplement: jiad316_Supplementary_Data [file jiad316_supplementary_data.zip › SupplementaryTable2.docx]

| Reference (first author, year, (trial name)), location | Participant details | **Total deaths** (deaths due to COVID-19) | | | | | **Total hospitalisations** (hospitalisations due to COVID-19) | | | |
| --- | --- | --- | --- | --- | --- | --- | --- | --- | --- | --- |
|  |  | **BCG** | | **Control** | | **BCG** | | | **Control** | |
| Czajka, 22,  Poland | Healthcare workers >25 years; mean age 46y  (n=354) | **0**^a^ | (0) | **0**^a^ | (0) | **unknown**^b^ | | (unknown) | **unknown^b^** | (unknown) |
| Dos Anjos, 22, Brazil | Healthcare workers with previous BCG (and scar); mean age 43y  (n=138) | **0** | (0) | **0** | (0) | **3** | | (1) | **0** | (0) |
| ten Doesschate, 22, BCG-CORONA,  Netherlands | Healthcare workers; mean age 42y  (n=1511) | **1** | (0) | **0** | (0) | **11** | | (1) | **17** | (2) |
| Upton, 22,  South Africa | Healthcare workers; median age 39y  (n=1000) | **0** | (0) | **4** | (2) | **30**^c^ | | (10) | **26^c^** | (5) |
| Tsilika, 22, ACTIVATE-2,  Greece | Adults >50y with significant comorbidity; mean age 69y  (n=301) | **0** | (0) | **3**^d^ | (unknown) | **unknown** | | (2) | **unknown** | (6) |
| Faustman, 22,  USA | Adults 18-50y with type 1 diabetes mellitus; median age 44y  (n=144, randomisation ratio 2 BCG:1 placebo) | **0** | (0) | **0** | (0) | **unknown** | | (unknown) | **unknown** | (unknown) |
| Moorlag & Taks, 22, BCG-CORONA-ELDERLY, Netherlands | Adults >60y; median age 67y  (n=2014) | **2** | (1) | **2** | (0) | **29** | | (1) | **16** | (2) |
| Sinha, 22, BRIC,  India | Adults 18-60y with significant comorbidity; mean age 44y  (n=495) | **unknown** | (0) | **unknown** | (1) | **unknown** | | (0) | **unknown** | (6) |
| Koekenbier, 23, BCG-PRIME, Netherlands | Adults >60y with comorbidity; median age 69y (n=6112) | **13** | (5) | **18** | (6) | **unknown^e^** | | (18) | **unknown^e^** | (21) |
| Santos, 23, ProBCG, Brazil | Healthcare workers (n=278) | **0** | (0) | **0** | (0) | **0** | | (0) | **0** | (0) |
| Pittet & Messina, 23, BRACE, Australia, Brazil, Netherlands, Spain, UK | Healthcare workers; mean age 43y  (n=3988) | **0** | (0) | **2** | (1) | **56^f^** | | (5^g^) | **36^f^** | (5^g^) |

**Supplementary Table 2 – Number of deaths and hospitalisations reported in each trial**

^a^ no comment was made on mortality in the trial, presumed no deaths.

^b^ one hospitalisation due to COVID-19 described, treatment group not stated.

^c^ participants may have experienced more than one event.

^d^ not stated whether these deaths were COVID-related.

^e^ 26 RTI-related hospital admissions in BCG group and 29 in placebo group, but total hospitalisations not reported.

^f^ non-elective admissions, first hospitalisation per participant only (uncensored data).

^g^ censored as per primary outcome analysis.

Randomisation ratio was 1:1 unless stated.
