# Supplementary material for: Interpreting the Results of Trials of BCG Vaccination for Protection Against COVID-19
Source: J Infect Dis. 2023 Aug 10;228(10):1467–78. doi: 10.1093/infdis/jiad316 (PMC10640778; doi:10.1093/infdis/jiad316)
Supplement: jiad316_Supplementary_Data [file jiad316_supplementary_data.zip › SupplementaryTable3.docx]

| Reference (first author, year, (trial name)), location | Domain 1: risk of bias arising from randomisation process | Domain 2: risk of bias due to deviation from intended interventions | Domain 3: risk of bias due to missing outcome data | Domain 4: risk of bias in measurement of the outcome | Domain 5: risk of bias in selection of the reported result | Overall risk of bias judgement | Key issues  (risk of bias domain: reason for concern) |
| --- | --- | --- | --- | --- | --- | --- | --- |
| Czajka, 22,  Poland | Low | Some concerns | Low | Some concerns | Some concerns | Some concerns | 2: Lack of information on analysis methods;  4: Participant-reported outcome (COVID-19 testing organised by participant);  5: Outcomes changed from clinicaltrials.gov entry (including primary outcome) and no protocol/ SAP available |
| Dos Anjos, 22, Brazil | Low | Some concerns | Low | Some concerns | Some concerns | Some concerns | 2: Analysis type (e.g. ITT) not specified and exclusions made post-randomisation;  4: Participant-reported outcome (COVID-19 testing organised and reported by participant);  5: Protocol available but analysis plan is not detailed. Vaccine efficacy was main measure presented but not described in protocol |
| ten Doesschate, 22, BCG-CORONA,  Netherlands | Low | Low | Low | Some concerns | Low | Some concerns | 4: Participant-reported outcome (work absence); |
| Upton, 22,  South Africa | Low | Low | Low | Low | Low | Low | n/a |
| Tsilika, 22, ACTIVATE-2,  Greece | Some concerns | Low | Low | Some concerns | Some concerns | Some concerns | 1: No detail of randomisation process;  4: Participant-reported outcome (symptom reporting, COVID-19 testing organised and reported by participant);  5: No protocol and registration page entry is brief, unclear if analysis plan changed |
| Faustman, 22,  USA | Low | Low/ some concerns | Low | Some concerns | Some concerns | Some concerns | 2: Lack of information on analysis methods;  4: Participant-reported outcome (symptom reporting);  5: No protocol available and clinicaltrials.gov updated after parallel study ended, therefore analysis plan unclear |
| Moorlag & Taks, 22, BCG-CORONA-ELDERLY,  Netherlands | Low | Some concerns | Low | Some concerns | Some concerns | Some concerns | 2: Analysis type (e.g. ITT) not specified and exclusions made post-randomisation;  4: Participant-reported outcome (symptom reporting);  5: Primary outcome changed after protocol published (and clinicaltrials.gov not updated) therefore analysis plan unclear |
| Sinha, 22, BRIC, India | Low | Low | Low | Some concerns | Some concerns | Some concerns | 4: Participant-reported outcome (symptom reporting), lack of detail on relevant symptoms/ test availability;  5: No protocol and registration page entry is brief, unclear if analysis plan or primary outcome definition changed |
| Koekenbier, 23, BCG-PRIME, Netherlands | Low | Low | Low | Some concerns | Low | Some concerns | 4: Participant-reported outcome (symptom reporting, COVID-19 testing organised and reported by participant), lack of detail on relevant symptoms/ test availability |
| Santos, 23, ProBCG, Brazil | Low | Some concerns | Low | Some concerns | Some concerns | Some concerns | 2: Analysis type (e.g. ITT) not specified and exclusions made post-randomisation;  4: Participant-reported outcome (COVID-19 testing organised by participant);  5: Protocol available on clinicaltrials.gov but analysis plan is not detailed. |
| Pittet & Messina, 23, BRACE, Australia, Brazil, Netherlands, Spain, UK | Low | Low | Low | Some concerns | Low | Some concerns | 4: Participant-reported outcome (symptom reporting, COVID-19 testing organised and reported by participant); |

**Supplementary Table 3 – Risk of bias judgements for the primary outcome of each trial (using Revised Cochrane risk-of-bias tool for randomized trials (RoB 2))**

Overall risk of bias judgement: low – the study is judged to be at low risk of bias for all domains for this result; some concerns – the study is judged to raise some concerns in at least one domain for this result, but not to be at high risk of bias for any domain; high – the study is judged to be at high risk of bias in at least one domain for this result, or the study is judged to have some concerns for multiple domains in a way that substantially lowers confidence in the result. Note: Participants were considered to be aware of treatment allocation group due to predictable skin reaction from BCG. Participants were also considered to be unblinded outcome assessors if primary outcome included participant-reported outcomes, or outcomes that were influenced by participant behaviour (symptoms, work absence and results of testing initiated by participants if they experienced symptoms). Hospitalisation was considered to be an objective outcome.
