# Supplementary material for: Interpreting the Results of Trials of BCG Vaccination for Protection Against COVID-19
Source: J Infect Dis. 2023 Aug 10;228(10):1467–78. doi: 10.1093/infdis/jiad316 (PMC10640778; doi:10.1093/infdis/jiad316)
Supplement: jiad316_Supplementary_Data [file jiad316_supplementary_data.zip › SupplementaryTable4.docx]

| Location  Trial name  (Registration details) | Participant details | Total  enrolled | Intervention (BCG strain) | Control intervention | Follow-  up duration | Primary outcome  S*econdary* *outcome, if COVID-19 cases not reported as primary outcome and results known* | Cases in BCG  group | Cases in control group | Reported summary measure |
| --- | --- | --- | --- | --- | --- | --- | --- | --- | --- |
| **India**  (NCT04475302 & CTRI/2020/06/ 025854) | 60-80y | 2175 | Moscow  SII | None | 6m | Mortality due to COVID-19 | † | † | † |
| **Denmark**  **BCG-DENMARK-SENIOR**  (NCT04542330 & EUCTR2020-003904-15-DK) | >65y | 1700 | Danish  1331, AJV | Saline | 12m | Acute infections requiring medical attention | † | † | † |
| **Denmark**  (NCT04373291 & EUCTR2020-001888-90-DK) | HCW | 1221 | Danish  1331 | Saline | 6m | Duration of unplanned absenteeism  for any illness (mean/ 1000 workdays) | 20 days^a^ | **17 days**^a^ | † |
|  |  |  |  |  |  | *COVID-19 cases*  *(antigen/ serology test)* | *43 (7.0%)*^a^ | ***33 (5.4%)***^a^ | † |
| **Guinea-Bissau, Mozambique**  **BCG-COVID-RCT**  (NCT04641858) | HCW | 668 | Danish,  1331, AJV | Saline | 6m | Days of unplanned absenteeism due to illness | † | † | † |
| **India**  (CTRI/2020/04/  024833)* | HCW | estimated 1826 | Danish | Saline | 6m | COVID-19 cases  (symptomatic with PCR/ serology) | † | † | † |
| **USA**  **BADAS**  (NCT04348370) | HCW | estimated 1800 | Tice | Saline | 6m | COVID-19 cases | † | † | † |
| **France**  **COVID-BCG**  (NCT04384549 & EUCTR2020-001678-31-FR)* | HCW | estimated 1120 | Danish  AJV | Saline | 6m | COVID-19 cases  (symptomatic with PCR/ serology/ imaging) | † | † | † |
| **Hungary**  (EUCTR2020-  001783-28-HU)* | HCW | estimated 1000 | Danish  1331, SSI | Saline | 6m | Days of absenteeism due to documented COVID-19 | † | † | † |
| **Mexico**  (NCT04461379)* | HCW | estimated 908 | Tokyo-172 | Saline | 6m | COVID-19 cases  (symptoms with serology) | † | † | † |
| **Egypt**  (NCT04350931)* | HCW | estimated 900 | Danish  1331 | Saline | 9m | COVID-19 cases  (confirmed) | † | † | † |
| **Brazil**  (NCT05507671) | Healthy adults | estimated 556 | Strain unspecified | Solvent of BCG vaccine | 6m | COVID-19 cases  (serology or symptoms) | † | † | † |
| **Iran**  (**IRCT202004110**  **47019N1)*** | HCW | estimated 500 | Strain unspecified | Saline | 12m | COVID-19 cases  (symptoms with PCR/ serology) | † | † | † |
| **India**  (CTRI/2020/09/  027684)* | HCW | estimated 400 | Strain unspecified | ? | 6w | COVID-19 cases | † | † | † |

**Supplementary Table 4 – Registered, unpublished trials of BCG to protect against COVID-19**

Trials believed to have either been completed (or due for completion) but results not yet published. Trials identified in searches of clinicaltrials.gov (https://clinicaltrials.gov, last search on 5/1/23) and World Health Organization International Clinical Trials Registry Platform (https://trialsearch.who.int, last search on 6/1/23).

* trial registration page not updated in >2 years.

† - primary outcome results not found.

^a^ results available at https://www.clinicaltrialsregister.eu/ctr-search/trial/2020-001888-90/results.

CI – confidence interval; HCW – healthcare worker; HR – hazard ratio; m – months; SII – Serum Institute of India; w – weeks; y – years.
